# The Synergy between Deep Learning and Organs-on-Chips for High-Throughput Drug Screening: A Review

**DOI:** 10.3390/bios13030389

**Published:** 2023-03-15

**Authors:** Manna Dai, Gao Xiao, Ming Shao, Yu Shrike Zhang

**Affiliations:** 1 College of Physics and Information Engineering, Fuzhou University, Fuzhou 350108, China; 2 Computing and Intelligence Department, Institute of High Performance Computing (IHPC), Agency for Science, Technology and Research (A*STAR), 1 Fusionopolis Way, #16-16 Connexis, Singapore 138632, Singapore; 3 College of Environment and Safety Engineering, Fuzhou University, Fuzhou 350108, China; 4 Department of Biomedical Engineering, Tsinghua University, Beijing 100084, China; 5 Department of Computer and Information Science, College of Engineering, University of Massachusetts Dartmouth, North Dartmouth, MA 02747, USA; 6 Division of Engineering in Medicine, Department of Medicine, Brigham and Women’s Hospital, Harvard Medical School, Cambridge, MA 02139, USA

**Keywords:** organs-on-chips, microfluidic systems, deep learning, drug screening, human-on-chips

## Abstract

Organs-on-chips (OoCs) are miniature microfluidic systems that have arguably become a class of advanced in vitro models. Deep learning, as an emerging topic in machine learning, has the ability to extract a hidden statistical relationship from the input data. Recently, these two areas have become integrated to achieve synergy for accelerating drug screening. This review provides a brief description of the basic concepts of deep learning used in OoCs and exemplifies the successful use cases for different types of OoCs. These microfluidic chips are of potential to be assembled as highly potent human-on-chips with complex physiological or pathological functions. Finally, we discuss the future supply with perspectives and potential challenges in terms of combining OoCs and deep learning for image processing and automation designs.

## 1. Introduction

Current drug research and development have faced the dilemma of long durations, large investments, and low rates of success. Preclinical drug development usually involves testing in static, planar cell cultures and animal models. However, conventional cell culturing oftentimes cannot reproduce the complex physiology and pathology of the human body, and animal models have drawbacks, such as species differences, high cost, low throughput, and ethics [1,2]. For example, patient-derived xenografts (PDXs) directly transplant tumor tissues from patients to immunocompromised mice without culturing, and hence, the biological specificities of the tumors are maintained to the greatest extent. However, the PDX models have very low success rates of transplantation. In addition, the applications of animal models are subject to the associated high costs, low throughput, and ethical issues in the early stages of drug discovery [3,4]. These reasons lead to a great risk of failure in human clinical trials of candidate compounds. Although significant progress has been made in computational biology, in vitro biology, and toxicology, most drugs have still failed to pass clinical trials due to the lack of efficacy and the problem of unwanted toxicity [5].

To provide effective alternatives for drug screening at the preclinical stage, the concept of microcell culture analogs (microCCAs) was initially proposed [6], which later on evolved into the terminology of organs-on-chips (OoCs) or microphysiological systems (MPSs) [7].

The OoC is a miniature device for dynamic three-dimensional (3D) cell culturing, and they have the merits of streamlined operations and small volumes. The OoC simulates the environment of the target human organ on the chip in order to study and control the biological behaviors of cells in the process of culturing in vitro. Although the OoCs may not completely replace animal experiments in most scenarios, they play an increasingly important role in the fields of toxicity assessment, disease modeling, and drug screening, among others [8].

OoCs have the strong advantages of rapid responses and desirable throughput and thus generate massive data. Researchers with biomedical backgrounds may find it difficult to manually analyze these data in short periods. Consequently, it is urgent to develop an automated tool that can assist or even replace researchers in conducting data analysis so as to improve the efficiency and accuracy of the experiment. Artificial intelligence (AI) [9] has strong abilities in feature representation and data mining, thereby achieving remarkable success in computer vision [10], text recognition [11], and natural language processing [12]. Nowadays, deep learning of AI has started to be applied to device design, real-time monitoring, and image processing in OoCs [2]. The integration of deep learning and OoCs offers a powerful tool for the exploration and analysis of massive image-based data, which consequently enhances the intelligence of OoCs and stimulates their great potential in higher-throughput drug screening.

**Figure 1:**
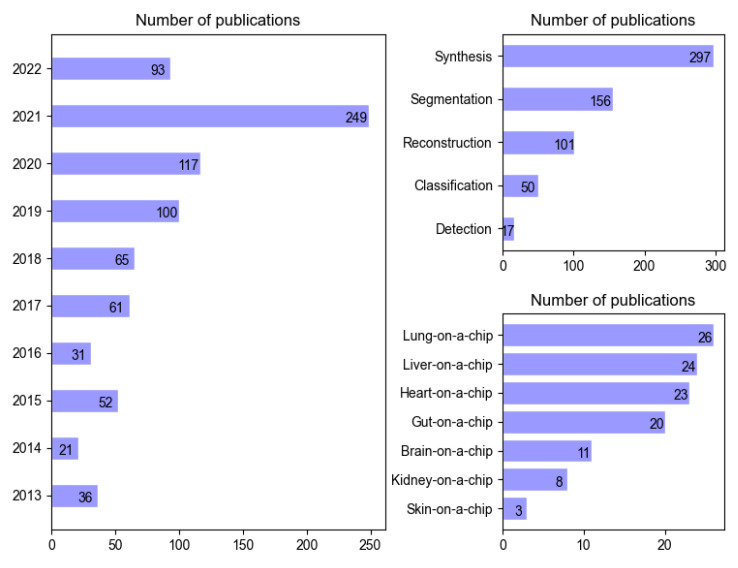
Breakdown of the publications included in this review according to the year of publication, task addressed in deep learning (Section 3), and application cases (Section 4). The number of publications for 2022 has been extrapolated from the publications published in or before April.

To provide a comprehensive overview of all relevant applications of deep learning and OoCs in higher-throughput drug screening, we used Google Scholar to search papers published in journals, conferences, and ArXiv in the past 10 years (2013–2022), including deep learning methods applied to different tasks, such as synthesis, segmentation, reconstruction, classification, and detection. We divided the reviewed papers into 7 categories according to the following applications: lung-on-a-chip, liver-on-a-chip, heart-on-a-chip, gut-on-a-chip, brain-on-a-chip, kidney-on-a-chip, and skin-on-a-chip. Descriptive statistics of these papers based on years, tasks, and practical cases can be found in Figure 1.

In summary, with this review, we aim to:

- Show that deep learning has begun to be explored in OoCs for higher-throughput drug screening.
- Highlight the critical deep learning tasks in OoCs and the successful use cases that solve or improve the efficiency of drug screening in the real world.
- Describe the potential applications and future challenges between deep learning and OoCs.

The remainder of the paper is structured as follows. We begin with a brief introduction of the principles of deep learning and widely used network structures in Section 2. Image-processing tasks based on various deep learning methods are described in Section 3. Section 4 summarizes existing examples where different deep learning methods are applied to OoC systems. Section 5 discusses the prospective applications and the future challenges of deep learning in OoCs.

## 2. Overview of Deep Learning Methods

This section introduces the concepts, techniques, and architectures of deep learning methods widely applied in high-throughput drug screening, especially in biomedical applications and the microscopy field. The included deep learning methods are neural networks (NN) [13], deep neural networks (DNN) [14], convolutional neural networks (CNN) [15], recurrent neural networks (RNN) [16], generative adversarial networks (GAN) [17], and auto-encoder (AE) [18].

Based on the availability of label information, deep learning methods can be divided into supervised and unsupervised learning. In supervised learning, given a dataset ${D=\{x_{n},y_{n}\}}_{n=1}^{N}$ of N samples where x is the observation and y is the label, supervised learning methods generally aim to optimize a regressor and classifier. When we feed data into the general supervised model $\hat{y}=f\left(x;W,B\right)$, we try to minimize the loss $L(y,\hat{y})$ between the predicted value $\hat{y}$ and ground truth value **y** and optimize the model parameters, including a set of weights $W=\{w_{1},w_{2},\cdots ,{w_{i},\cdots ,w}_{K}\},$ and a set of biases $B=\left\{b_{1},b_{2},\cdots ,{b_{i},\cdots ,b}_{K}\right\}$ during the training. In unsupervised learning, the dataset ${D=\{x_{n}\}}_{n=1}^{N}$ excludes the label information and focuses on tasks including clustering, dimensionality reduction and representation learning. For example, representation learning uses AE to minimize the reconstruction loss $L(x,\hat{x})$ between the original data x and the reconstructed one $\hat{x}$ to enable the encoder to learn the latent representation of the data in a lower-dimensional space.

### 2.1. NN and DNN

NN is the foundation of modern deep learning methods, as well as the state-of-the-art machine learning model since the 1980s. A typical NN consists of an input layer, one or more hidden layers, an output layer, and neurons within each layer. Each neuron connects to another one and has an associated activation a, a set of weights W and a set of biases B. At the final layer of the network, a probability of classification $P\left(y|x;W,B\right)$ is calculated by passing the activation through a softmax function.

(1) $$P\left(y|x;W,B\right)=softmax\left(x;W,B\right)=\frac{e^{w_{i}^{T}x+b_{i}}}{\sum_{k=1}^{K}e^{w_{k}^{T}x+b_{k}}},$$

where $w_{i}$ indicates the weight vector leading to the output neuron associated with the class y=i.

The probability function above is parameterized by W and B on dataset D. A common approach to solving the function is the maximum likelihood estimate (MLE) [19] with stochastic gradient descent, which, in practice, is equivalent to minimizing the negative log-likelihood [20]:

(2) $$arg\underset{\theta }{\min}-\sum_{n=1}^{N}\log\left[P\left(y_{n}|x_{n};W,B\right)\right].$$

The obtained softmax score will be further used in binary cross-entropy for binary classification and categorical cross-entropy for multiple classifications [21,22,23,24].

While NN models were invented decades before, issues such as the local optimum lead to poor performance and hard training. To that end, four strategies are widely utilized during training. (i) Mini-batch [25,26]: mini-batch only utilizes a batch of data instead of full data during each update to reduce memory usage and improve the training efficiency. (ii) Stochastic gradient descent (SGD) [27,28]: The SGD strategy adds random factors in gradient calculation, which is generally fast and benefits the model’s generalization. In addition, the randomness may help avoid local minimum and continue searching for the global minimum. (iii) Simulated annealing [29,30]: At each step, simulated annealing will accept a suboptimal solution with a probability that decays over iterations, which is another practical approach to avoiding the local minimum. (iv) Different initialization parameters [31]: This approach suggests initializing multiple neural networks with different parameter values and choosing the parameters with the smallest errors as the final solution.

### 2.2. CNN

CNN is a popular variation of DNN with convolutional layers inspired by the receptive field mechanism in biology. Compared to conventional DNN, CNN has two unique merits. First, the full connection architecture in DNN layers usually leads to parametric expansion, along with local optimum and vanishing gradient problems. CNN, on the other hand, mainly uses convolution layers, which drastically reduces the number of parameters to be learned through weight-sharing. Second, CNN and its convolution layers and pooling layers are particularly suitable for image feature learning or grid data in general. Convolution layers can maximize local information and retain plane structure information while the pooling layers (i.e., mean pooling and max pooling) aggregate the pixel values of neighborhoods via a permutation invariant function. This architecture allows for translation invariance and again reduces the number of weights in the CNN. Specifically, at Layer l, the k-th feature map $x_{k}^{l}$ is formulated as:

(3) $$x_{k}^{l}=\sigma \left(w_{k}^{l-1}{\times x}^{l-1}+b_{k}^{l-1}\right),$$

where $x^{l-1}$ is the output feature map at Layer l−1, and σ represents an element-wise non-linear transform function. The top layers of CNN are usually implemented as fully connected, and thus, weights are no longer shared. Similar to DNN, the activations at the last layer are fed to a softmax function to compute the probability of each class. The objective function of training is solved by MLE.

### 2.3. RNN

While CNN has been widely applied to grid data, e.g., 2D images, it fails to explicitly model the temporal changes over time in time series data. To that end, RNN establishes weight connections between neurons in each hidden layer, which allows the output at time t to be used as the input for time (t+1). Therefore, RNN is suitable for multi-variate time series, e.g., language translations, natural language processing [9], and video analysis where the input to RNN is a high-dimensional sequence ${\{x}_{1},x_{2},\cdots ,x_{T}\}$. Then, the hidden state $h_{T}$ over time T is passed through one or more fully connected layers. Last, the output will be fed into a softmax function [32] to calculate the probability of classification:

(4) $$P\left(y|x_{1},x_{2},\cdots ,x_{T};U,W,B\right)=softmax\left(h_{T};U,W,B\right),$$

where U represents the state-input weights of recurrent cells, W denotes the state–state weights of recurrent cells, and B is a set of biases.

While RNN is capable of modeling time-series data, it suffers from the long-term dependencies problem [33], resulting in gradient vanishing and gradient explosion. Follow-up solutions, e.g., leak unit (i.e., linear self-connection unit), partially addressed the issue but also have two deficiencies. One is that the manually set weights are not optimal in the memory system. The other is that the leak unit lacks a forgetting function and is prone to information overload. Therefore, a gated unit was introduced that is capable of forgetting the past states that are fully used by the recurrent cells. Successful implementations with gated units include long short-term memory (LSTM) [34] and gated recurrent unit networks (GRU) [35].

### 2.4. GAN

AI-generated content (AIGC) has been widely discussed recently, and one of the popular AIGC tools is GAN. In addition to content generation, e.g., artwork and style translation, GAN plays key roles in general data augmentation where data are relatively expensive to collect. Once properly trained, GAN is able to generate data under the same distribution but that did not exist before. These “high-fidelity” data can be used as additional training data in addition to the augmentation by rotation, crop, and varying illumination.

The vanilla GAN is a generative model that conducts direct sampling or inference from the desired data distribution without the Markov chain learning mechanism [36]. The GAN consists of two NNs: the generator G and the discriminator D. Two networks compete and eventually reach a balance when G receives random noise and generates data $x_{g}$ that D fails to distinguish from the actual data $x_{r}$. The training objectives of G and D is a “min-max” game between their respective loss functions. Essentially, D is trying to detect the forged area, and hence D maximizes the loss function $L_{D}$:

(5) $$L_{D}=\underset{D}{\max}E_{x_{r}\sim p_{r}\left(x\right)}\left[\log D\left(x_{r}\right)\right]+E_{x_{g}\sim p_{g}\left(x\right)}\left[\log\left(1-D\left(x_{g}\right)\right)\right].$$

Once D’s learning is finished, D is fixed, and G training starts. Since G aims to generate the data under the same distribution, its training minimizes the following:

(6) $$L_{G}=\underset{G}{\min}E_{x_{g}\sim p_{g}\left(x\right)}\left[\log\left(1-D\left(x_{g}\right)\right)\right].$$

Overall, D and G’s networks are trained alternately until converged. In general, GAN is adopted for data generation or unsupervised learning [37]. Recent work has proposed adding a gradient penalty [23] to the critic loss to avoid the problems of exploding and vanishing gradients in GAN.

### 2.5. AE

Representation learning has recently been playing an increasingly important role in pretraining, thanks to the cheap unlabeled data. Among them, AE is one of the most fundamental models that learn in an unsupervised manner. AE uses an encoder to map the input data x into a latent vector and has a decoder to reconstruct the input data $\hat{x}$ from the latent vector. Since the dimension of the latent vector is usually small, the latent vector is usually treated as features or learned representation with compression.

For an encoder with a hidden layer, the input data are passed through a non-linear function, which is formulated as:

(7) $$z=f\left(W_{1}x+B_{1}\right),$$

where z stands for the latent vector, f denotes the non-linear function of the encoder, $W_{1}$ represents the weight matrix, and $B_{1}$ is the bias matrix. Then, the latent vector is fed to the decoder, which contains a hidden layer:

(8) $$\hat{x}=g\left(W_{2}z+B_{2}\right)$$

where $\hat{x}$ stands for the reconstructed input, g denotes the non-linear function of the decoder, $W_{2}$ represents the weight matrix, and $B_{2}$ is the bias matrix. The parameters of the AE are optimized by minimizing the mean square error (MSE) loss function [38], equivalent to minimizing the differences between decoder output $\hat{x}$ and the encoder input x.

There are takeaways regarding the usage of AE. First, AE is data-specific, or in other words, data-dependent, meaning the efficacy of compression depends on the similarity to the training datasets. Second, the AE conducts lossy compression, and the output of its decoder is degraded compared to the original input. Third, AE learns from training datasets regardless of labels. However, when labels are available, class-specific encoders can be learned without additional work. Last, AE is mainly used for unsupervised pretraining followed by supervised fine-tuning [24] to resolve the problem of initializing weights, vanishing gradients, and model generalization.

## 3. Deep Learning Methods Potentially Useful for OoCs

Several key technologies arise from the various OoCs, which are categorized into 5 canonical tasks: synthesis, segmentation, reconstruction, classification, and detection. Since the technical combination of deep learning and OoCs is at the proof of concept (PoC) so far, we provide the following application prospects for consideration.

### 3.1. Image Synthesis (Super-Resolution, Data Augmentation)

**Figure 2:**
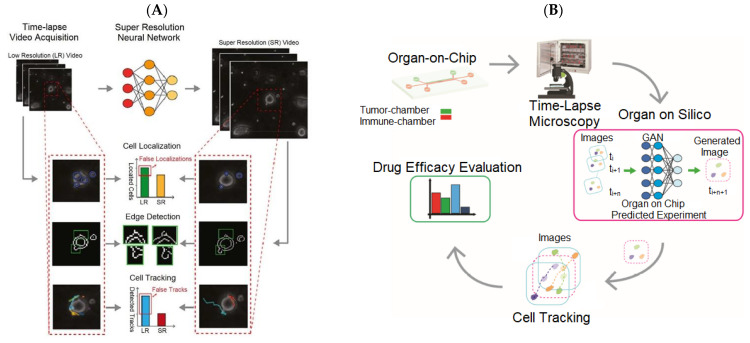
Application of DL in TLM videos for improving the accuracy rate of detecting cell migrations and interactions in OoC experiments. (**A**) Super-resolution method for TLM video frames. This method utilizes un-trained NN to obtain super-resolved images while fitting the input low-resolution video frames without paired training data [41]. Reproduced with permission from Elsevier Copyright (2023). (**B**) Data augmentation for TLM videos. The proposed method generates interleaved video frames for providing high-throughput TLM videos. These two methods can effectively improve the accuracy of cell tracking [43]. Reproduced with permission from Springer Nature Copyright (2023).

Image synthesis is one of the first areas in which deep learning made a major contribution to the field of OoCs. Biological experiments based on OoCs oftentimes utilize light-based time-lapse microscopy (TLM) to observe cell movements and other structural alterations, and a high spatial resolution is critical for capturing cell dynamics and interactions from data recorded by the TLM [39]. However, due to the high costs of advanced devices, high-resolution images and videos are not always acquired. To improve the image resolution, we [40] trained a GAN model to enhance the spatial resolution of mini-microscopic images and regular-microscopic images acquired with different optical microscopes under various magnifications. To address the issue of video resolution, Pasquale Cascarano et al. [41] extended the deep image prior (DIP) [42] in image super-resolution to the recursive deep prior video (RDPV) for video frames so as to improve the spatial resolution of TLM videos. The author of the DIP demonstrated that a randomly initialized CNN could be used as a hand-crafted prior with excellent results in a super-resolution task. Based on this, the same prior could also be adopted for restoring images for which paired training data were hard to collect. Instead of searching for the answer in the image space, the DIP searched in the space of the CNN’s parameters. The DIP was utilized to fit a low-resolution image, which converted a super-resolution task to a conditional image generation problem. The needed information for CNN’s parameter optimization were low-resolution images and the hand-crafted prior produced by the CNN. Similar to DIP, the utilized CNN architecture in the RDPV was built as an encoder-decoder framework. The RDPV was fed with one low-resolution frame from a TLM video at a time and applied the knowledge of previous super-resolved frames to reconstruct the new one through a recursively updating the weights of the CNN. Figure 2A depicts an example of video frame reconstruction with RDPV. When using the TLM video improved by the RDPV, the researchers can effectively decrease the error of cell localization, successfully detect the clear edges of cells, and draw a precise trajectory for cell tracking.

In addition, when observing the cell movements and cell–cell interactions, it is desirable for the TLM to increase the frame rate for accurately reconstructing cell-interaction dynamics. However, high frame rates increase photobleaching and phototoxicity so as to affect cell growth and imaging quality. The balance between high-resolution and carried information content is required to reduce the overall data volume. Comes et al. [43] built a multi-scale GAN to generate interleaved frames of the predicted cell moving and inserted them into the original videos to provide high-throughput videos. This GAN architecture not only increased the temporal resolution of original videos but also preserved the biological information in the original videos. Figure 2B shows the flowchart of work [44].

### 3.2. Image Segmentation

**Figure 3:**
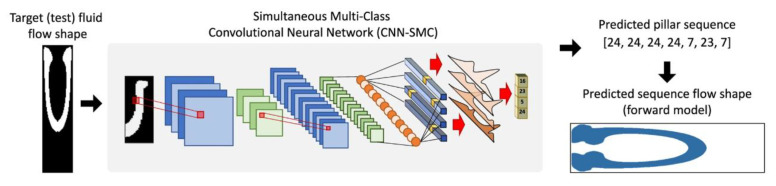
Application of deep learning for nerve cell segmentation. This photograph is directly cropped from the corresponding papers [44]. Reproduced with permission from Springer Nature Copyright (2023).

Some OoC experiments need to segment the cell populations from the images for different analyzing tasks. Stoecklein and colleagues [44] utilized a CNN to segment nerve cell images into three categories consisting of the axon (blue), myelin (red), and background (black). As shown in Figure 3, a target fluid flow shape was input to the CNN, which outputs a predicted pillar sequence. This predicted pillar sequence was fed into a forward model to predict the sequence flow shape, which was compared with the original target fluid flow shape by computing the pixel match rate (PMR) [45].

The U-Net [46,47,48] was successfully applied in various image segmentation tasks, especially for cell detection and shape measurements in biomedical images. The authors [49] developed a plug-in for the ImageJ software [50] to conduct a flexible single-cell segmentation. This plug-in can produce the segmentation mask from an input cell image.

### 3.3. Image Reconstruction

Lim et al. [51] reconstructed all pixels of red blood cells (RBCs) [52] by using a DNN-based network, which greatly eliminates the introduced distortions due to the ill-posed measurements acquired from the limited numerical apertures (NAs) [53] of the optical system. This network has been validated to exactly compute the 2D projections for reconstructing the 3D refractive index distributions.

### 3.4. Image Classification

**Figure 4:**
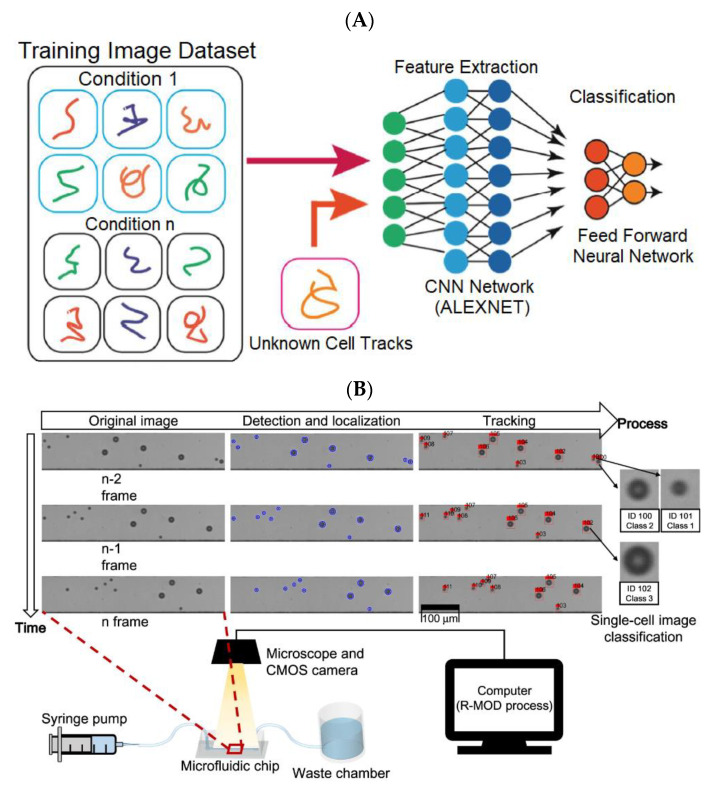
Application of deep learning in classification. A and B are directly cropped from the corresponding papers [54,58], respectively. (**A**) The work [54] utilized AlexNET to classify the cell motility behaviors by implementing transfer learning on the input cell trajectories. Reproduced with permission from Springer Nature Copyright (2023). (**B**) Schematic of the designed system and the real-time moving object detector (R-MOD) in work [58]. Reproduced with permission from Springer Nature Copyright (2023).

Classification is one of the most widely used technologies in deep learning. The image labels are adopted to train a classifier, which can successfully extract hierarchical image features. In Figure 4A, Mencattini et al. [54] developed a CNN (AlexNET) [55] to perform experimental classification on an atlas of cell trajectories via a predefined taxonomy (e.g., drug and no-drug). They reposted that the cell trajectories were detected from the video sequences acquired by the TLM in a Petri dish [56] or in an OoC platform [54]. This method was able to accurately classify single-cell trajectories according to the presence or not of the drugs. This method was inspired by the successful application of deep learning for style recognition in paintings and artistic style transfer [57]. This method reveals the universal motility styles of cells, which are identified by deep learning in discovering unknown information from cell trajectories.

Because of motion blur, it is extremely difficult to acquire a high-quality image of a flowing cell. To address it, the researchers [58] proposed to construct high-throughput imaging flow cytometry (IFC) by integrating a specialized light source and additional detectors with conventional flow cytometry (FC) [59] (Figure 4B). The complementary metal-oxide semiconductor (CMOS) camera [60] on the microscope collected image sequences of the microfluidic channel through which cell suspension flowed. The multi-tracking technology was utilized for the original region-of-interest (ROI) image frame so as to crop the single-cell images from the video sequence. The cropped single-cell images were passed to a classifier based on supervised learning to identify the cell type. Since multiple cells could be detected and tracked simultaneously, the proposed method could maintain high throughput at a low flow rate by increasing the concentration of cells.

### 3.5. Image Detection

To understand the anatomic and dynamic properties of cells, it is necessary to analyze the massive amounts of time-lapse image data of live cells to this end. Tracking large numbers of cells is a common method to analyze the dynamic behavior of cell clusters. On a tumor-on-a-chip device [2], CellHunter [61] was proposed for tracking and motion analysis of cells and particles in time-lapse microscopy images. By using CellHunter, the effective movement of dendritic cells toward tumor cells was assessed.

Currently, most detection methods are based on supervised or semi-supervised learning and need tremendous datasets with labels or annotations. However, the process of labeling training images is largely manual, which is time-consuming. Some unsupervised learning approaches without manual annotations are proposed to tackle this limitation. The authors [62] studied the OoC for the culture of complex airway models. They built connections between microscopic and macroscopic associated objects by embedding the fuzzy C-means cluster algorithm [63] into the cycle generative adversarial network (Cycle GAN) [64]. This network took advantage of transfer learning for toxoplasma detection and achieved high accuracy and effectiveness in toxoplasma microscopic images.

## 4. Case Studies in OoC Applications

**Figure 5:**
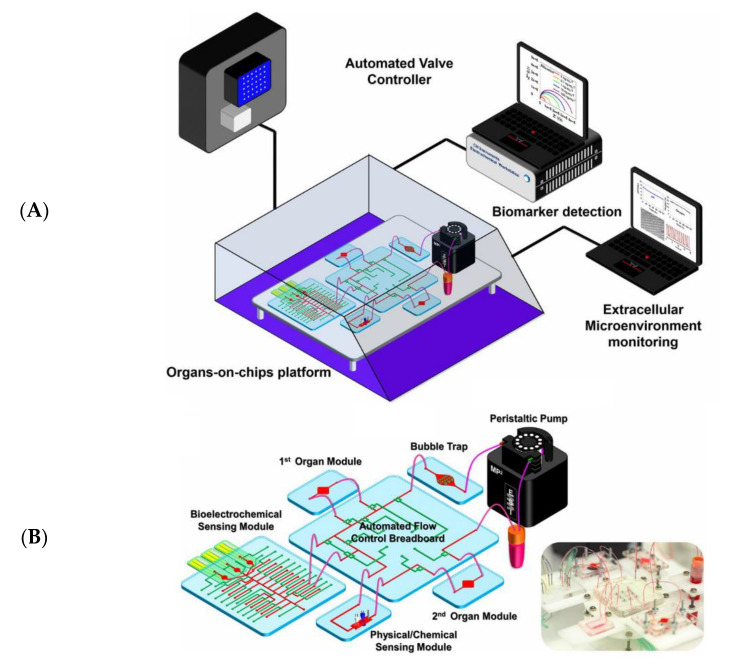
The idea of an automated monitoring and analysis platform integrating multiple OoCs with sensors for maintaining appropriate temperature and CO_2_ levels [66]. (**A**) The schematic of a multi-OoC platform in a benchtop incubator, which is connected with automated pneumatic valve controller, electronics for operating physical sensors, potentiostat for measuring electrochemical signals, and a computer for central programmed integration of all commands. (**B**) The in-house designed multi-OoC platform contains a breadboard, microbioreactors, medium reservoir, a physical sensing suite, one or multiple electrochemical sensors, and bubble traps. Reproduced with permission from Proceedings of the National Academy of Sciences Copyright (2023).

Table 1 provides a summary of representative applications of deep learning used for different OoCs. Although at a very early stage and hence with limited demonstrations to date, the combination of OoCs and deep learning represents a breakthrough for drug screening and related applications [65]. Given the appropriate data quantity and data quality, deep learning approaches can potentially be used throughout the drug screening pipeline to reduce attrition. In addition, OoCs with AI boost the capacity for high-throughput drug screening and, to some extent, reduce the ethical and legal regulation problems in animal models due to the possibility of avoiding some animal experiments. Figure 5A depicts a full system that integrates OoCs with multi-sensors for automatically monitoring microtissue behaviors [66]. The data acquired from physical/chemical and electrochemical sensing modules are analyzed by AI modules, which are designed for image processing, signal abnormal diagnosis, data classification, and prediction. This multi-sensor information fusion was not previously available but nowadays can be applied for potentially enhancing the efficiency of drug screening. The detailed structure of the integrated multi-OoCs is provided in Figure 5B, including microbioreactors for housing organoids, a breadboard for microfluidic routing via pneumatic valves, a reservoir, bubble traps, physical sensors for measuring microenvironment parameters, and electrochemical biosensors for detecting soluble biomarkers secreted by the microtissue.

### 4.1. Lung-on-a-Chip

**Figure 6:**
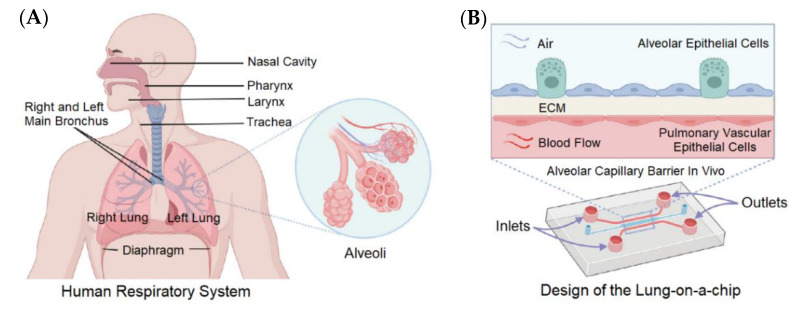
Alveolar–capillary barrier in vivo mimicked in a lung-on-a-chip model [78]. (**A**) The exchange of O_2_ and CO_2_ occurs in the human lungs, especially in the alveoli. (**B**) Cross-section of the lung model on microfluidic chip, where two different channels are separated by a thin, porous membrane. Reproduced with permission from Elsevier Copyright (2023).

There is a pressing need for effective therapeutics for coronavirus disease 2019 (COVID-19), which is a respiratory disease caused by the severe acute respiratory syndrome coronavirus 2 (SARS-CoV-2) virus [75,76,77]. The SARS-CoV-2 virus affects several tissues, including the lung, where the unique 3D structure of its functional units is critical for proper respiratory function. The lung-on-a-chip is an in vitro lung model, which essentially recapitulates the distinct tissue structure and the dynamic mechanical and biological interactions between the different cell types. Figure 6 depicts the design of a lung-on-a-chip, which successfully replicates the physiology and pathology of the human lungs for culturing immortalized cell lines or primary human cells from patients [78]. As shown in the cross-section of the lung model of Figure 6B, human alveolar epithelial cells at the upper channel and human pulmonary microvascular endothelial cells at the lower channel were separated by the extracellular matrix (ECM)-coated membrane. Once confluent, the media was aspirated from the upper channel to cultivate alveolar cells at the air–liquid interface, and a syringe pump was connected to the lower channel to continuously infuse the media.

**Figure 7:**
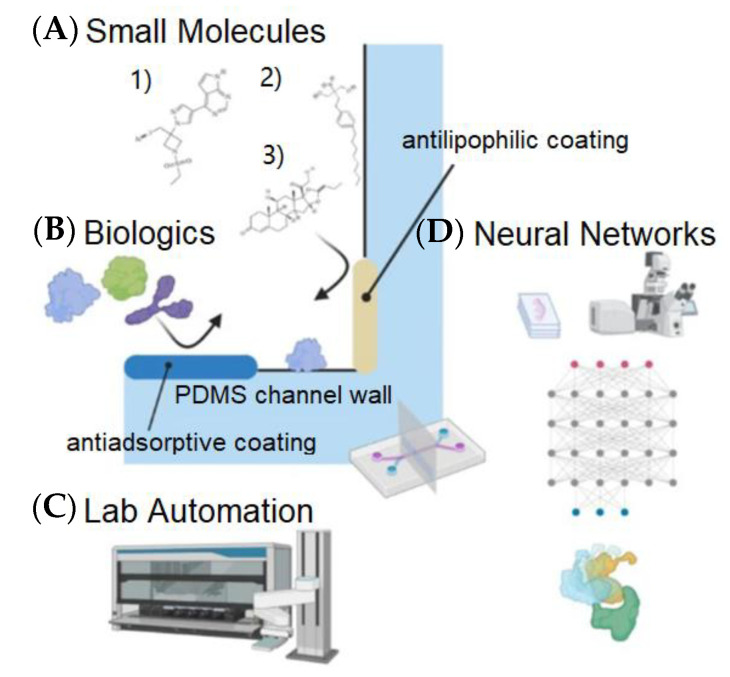
Application of deep learning in lung-on-a-chip and upcoming advances. This figure is directly reproduced from the corresponding paper [67]. (**A**) Small lipophilic molecules bind to surfaces such as PDMS channel walls and can be characterized by the Langmuir–Freundlich isotherm. (**B**) Biologics such as antibodies and recombinant proteins adsorb to PDMS channel walls. (**C**) Integrating lung-on-a-chip with automated liquid handling and continuous flow. (**D**) AI algorithms such as NNs can aid drug discovery through molecular docking and design, image analysis, and toxicity predictions. Reproduced with permission from Springer Nature Copyright (2023).

Deep learning can be introduced into the lung-on-a-chip to accelerate drug development for COVID-19 and beyond. Sun et al. [66] reported that the lung-on-a-chip with deep learning has been utilized in COVID-19 infection studies, which is depicted in Figure 7. In Figure 7A, small-molecule immunosuppressants can inhibit the JAK/STAT pathway intracellularly and have been suggested for use against COVID-19-associated HLH. These small molecules bind to PDMS channel walls. In Figure 7B, biologics adsorb to PDMS channel walls, and the antiadsorptive coating is a method to prevent adsorption. In Figure 7C, a lung-on-a-chip is integrated with automated liquid handling and continuous flow, which would provide a new solution for streamlining drug discovery and increasing throughput for screening lead compounds. In Figure 7D, deep learning algorithms (e.g., NNs) can aid drug discovery through molecular docking and design, image analysis, and toxicity predictions. Effective usage includes generating and seeking out sufficiently large datasets to train algorithms to make accurate predictions.

### 4.2. Liver-on-a-Chip

**Figure 8:**
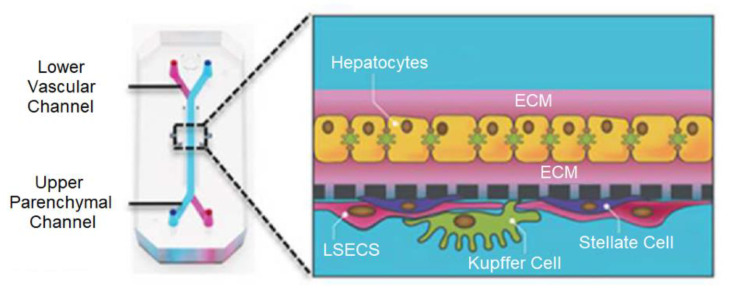
The cross-section of the liver-on-a-chip for simulating hepatic sinusoids [80]. Reproduced with permission from Elsevier Copyright (2023).

Drug-induced liver injury (DILI) is a major cause of drug failure [79]. Drug metabolism leads to bio-transformations of pharmaceutical substances that alter drug efficacy, toxicity, and drug interactions. The liver is the primary site of drug metabolism, but traditional liver models cannot replicate the complex physiological structure and microenvironment of the liver, especially the O_2_ and nutrient gradients. Therefore, many researchers are making efforts to develop the liver-on-a-chip and have achieved significant progress in relevant technologies. Figure 8 is a schematic of a liver-on-a-chip for recapitulating liver cytoarchitecture [80]. Primary hepatocytes were grown in the upper parenchymal channel with the ECM sandwich format, while the liver sinusoidal endothelial cells (LSECs), Kupffer cells, and hepatic stellate cells were populated in the lower vascular channel.

However, the field is still somewhat in its infancy in terms of the standards, procedures, and methods for translating the data obtained in vitro into reliable predictions applicable to human body responses [81]. Some deep learning methods were built to predict a chemical’s toxic potential in silico so as to replace in vitro high-throughput screening [82]. One example is the Tox21 project for toxicity assays, which is a database comprised of compounds with various activities in each of the 12 different pathway assays. To this end, Capuzzi et al. [83] built quantitative structure-activity relationship (QSAR) [84] models by using the random forest method [85], DNNs, and various combinations of molecular descriptors and dataset-balancing protocols. However, the large experimental dataset has a higher chance of containing mislabeling either the chemical structures or their toxicity classes. To expand the availability of highly confident data, industry-driven collaborative efforts are required. In addition, Li et al. [2] reported that Johnson & Johnson used the liver-on-a-chip to test the hepatotoxicity of drugs [86]. Zhang et al. [87] reported that introducing AI [88] into OoCs could effectively improve the ability of data analysis of biomedical platforms.

### 4.3. Heart-on-a-Chip

**Figure 9:**
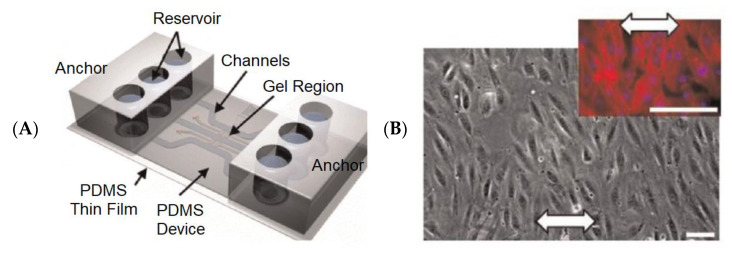
The heart-on-a-chip platform for culturing hMVECs [90]. (**A**) Schematic of the heart-on-a-chip. (**B**) Perpendicular alignment of hMVECs cultured in this heart-on-a-chip (10%, 1-Hz strain). Reproduced with permission from Elsevier Copyright (2023).

Heart diseases are the major killers threatening human health, and drug-induced cardiotoxicity is a major problem in drug development [89,90,91]. To resolve these two problems, many researchers are devoted to studying heart diseases in different manners. The heart-on-a-chip is a novel way of building heart models in vitro, and it is a promising tool for the study of heart diseases and drug screening. Figure 9A is the schematic of a heart-on-a-chip, including medium reservoirs, microfluidic channels, gel-loading ports, and a thin PDMS membrane within the PDMS device [90]. Figure 9B is a screenshot of human microvascular endothelial cells (hMVECs) cultured in this microfluidic system.

Two sensing methods are mainly employed in heart-on-a-chip for physical and electrical measurements [92]: (i) optical sensors, which are devices related to direct and calcium imaging, and fluorescent, laser-based, and colorimetric sensing; (ii) electrical sensors, which record the contractility of cardiomyocytes in real time, such as impedance, strain, and crack sensing. However, these electrical sensors have limitations on the number of recording sites and the capacity to process huge amounts of data. Hence, the sensors based on deep learning can be developed and introduced into the heart-on-a-chip for both optical and electrical-based measurements, to facilitate automated analysis, and to improve the accuracy of cardiac physical and electrical monitoring. In addition, the deep learning-based algorithms can acquire the physical properties (including size, shape, motility, and moving patterns) and electrophysiological features (such as strength, velocity, and propagation pattern of action potential) of numerous cells in order to increase the accuracy of predicting both therapeutic and unexpected side effects of novel drug candidates during drug screening [93,94].

### 4.4. Gut-on-a-Chip

**Figure 10:**
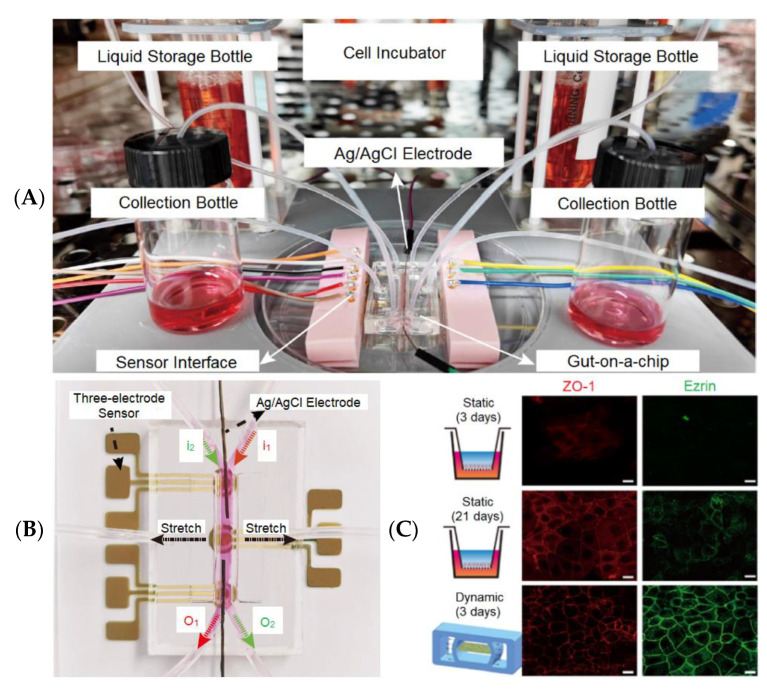
The gut-on-a-chip platform for exploring the transport mechanism of Hg(II) [68]. (**A**) The actual design of the gut-on-a-chip platform. (**B**) A photograph of the gut-on-a-chip connecting with multi-sensors. (**C**) A confocal fluorescence photograph of a tight junction protein (red-marked ZO-1) and brush border protein (green-marked ezrin) in static (3 days; 21 days) and dynamic cultures (3 days) (scale bar 20 μm). Reproduced with permission from Springer Nature Copyright (2023).

Many drugs are absorbed through the gut, and nowadays, the gut microbiome research community commonly utilizes laboratory mice to study drug performance on diseases. However, Marrero et al. [95] reported that animal models often failed when extrapolated to humans due to the complex gut dynamics, the interactions of the host and different microbiota components, and different immune systems between species. The latest gut-on-a-chip attempts to replicate the relationship between gut inflammation and host-microbial population so as to clarify the pathological mechanism of early intestinal diseases. Therefore, the gut-on-a-chip is a particularly necessary model to improve the knowledge of intestinal physiology and disease etiology [96]. Figure 10A is a full system integrating a gut-on-a-chip with its monitoring and culturing component [68]. Figure 10B shows the schematic of a gut-on-a-chip, which has the simultaneous integration of three-electrode sensors and an Ag/AgCl electrode for the in situ detection of Hg(II) and transepithelial electrical resistance (TEER). Figure 10C depicts the expression of the tight junction protein (ZO-1, red staining) and brush border protein (ezrin, green staining) in static culturing (3 days and 21 days) and dynamic culturing (3 days). The immunofluorescence staining of ZO-1 and ezrin demonstrated that Caco-2 cells displayed tight junctions and brush borders. The resolution of confocal fluorescence photographs can be enhanced by involving AI algorithms (GAN [97], CNN [98]) and can thus potentially conduct a better analysis of protein expression.

Shin et al. [99] reported gut-on-a-chip devices inhabited by microbial flora. To develop a high-throughput system, Trietsch et al. [100] reported a gut-on-a-chip array and demonstrated the efficiency of testing for drug toxicity. These multiplied gut-on-a-chip devices generated huge amounts of data, and hence deep learning technology is needed for data acquisition, data communication, and data analysis. During data acquisition and data communication, as many related sensors are involved, the novel visual sensor networks (VSNs) [101] can be used to perceive visual information (e.g., videos, images) in the ROI so as to improve the quality of data communication. A VSN contains a set of spatially distributed visual sensor nodes with the capabilities of image processing, communication, and storage [102]. The key technologies of image processing for improving the performance of a VSN are image segmentation and super-resolution reconstruction. Therefore, many state-of-the-art AI methods based on deep learning can be transplanted into multiplexed gut-on-a-chip devices. In addition, deep learning can also be integrated into the drug testing phase for predicting the effectiveness of the new drug and its side effects in the short and long term. Marrero et al. [95] proposed an alternative biosensing solution, which could translate to a gut-on-a-chip from other devices used in vitro or lab-on-a-chip.

### 4.5. Brain-on-a-Chip and Brain Organoid-on-a-Chip

**Figure 11:**
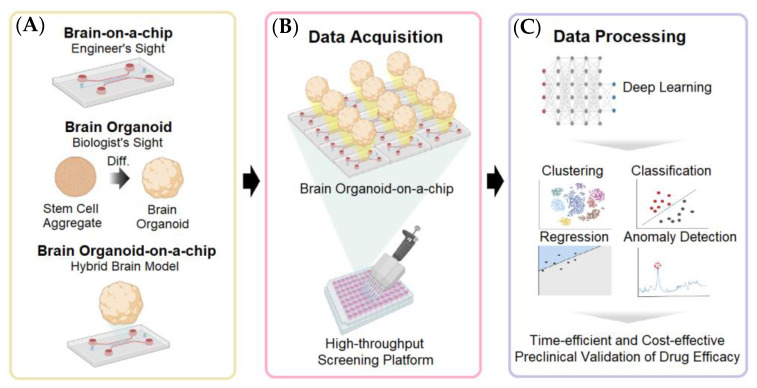
Comparison of human brain avatars and the deep learning techniques for high-throughput drug screening [104]. (**A**) The relationship between different brain avatars. (**B**) The injection-molded microfluidic chip allows the high-throughput drug screening of brain organoids-on-a-chip. (**C**) Deep learning is needed to conduct biological data analysis on massive data for high-throughput drug screening. Reproduced with permission from AIP Publishing Copyright (2023).

It is challenging to develop new drugs for treating neurodegenerative diseases and neurodevelopmental disorders due to the poor understanding of pathogenesis and the lack of appropriate experimental models. Animal models have drawbacks, including ethical concerns, genetic heterogeneity with humans, and high costs [1]. Brain-on-a-chip and brain organoids are two alternatives that have been extensively studied [103]. As shown in Figure 11A, brains-on-a-chip have been mainly developed in the field of engineering, which can construct sophisticated and complex microstructures for 3D cell cultures by using microfabrication techniques [104]. Brain organoids belong to the biological field. Cakir et al. [105] reported that vascularized brain organoids could be formed through the co-culturing of brain organoids and endothelial cells. Alternatively, certain portions of stem cells within the stem cell aggregates could be differentiated into brain endothelial cells. Although brain organoids have great potential to mimic the ultrastructure of the brain tissue, the brain-on-a-chip is good at reconstructing the characteristics of the brain microenvironment on the engineering platform. However, these two technologies also have limitations in the generalization of microenvironment characteristics and structures, which means that more in vivo-related brain models are needed. In this regard, brain organoid-on-a-chip has emerged to serve as a novel “human brain avatar”, which was formed by incorporating matured brain organoids into the brain-on-a-chip with hydrogels [106]. As shown in Figure 11B, brain organoid-on-a-chip has a heterogeneous 3D structure in a single organoid, and its unit size is large, which makes it difficult to image at high magnification. Therefore, continuous imaging should be performed to visualize the height-dependent structures, which is essential for high-content screening. In addition, for high-throughput screening, an automatic imaging system should be used to image multiple organoids. In both cases, it is too difficult to identify the number of massive images in a labor-intensive manner (Figure 11B). Therefore, deep learning techniques can be utilized for data analysis in both HCS and HTS, ranging from supervised learning methods (CNN, RNN) to unsupervised learning methods (deep generative models) [69]. These algorithms are capable of clustering, classification, regression, and anomaly detection (Figure 11C).

Deep brain stimulation (DBS) [107] is a surgical treatment for motor symptoms of Parkinson’s disease (PD) [108], which can provide electrical stimulation to the basal ganglia (BG) [109] region of the brain. Existing commercial DBS devices only use stimulation based on fixed frequency periodic pulses, but this device is very inefficient in terms of energy consumption. Moreover, fixed high-frequency stimulation may have side effects, such as speech impairment. To address the above problems, Gao et al. [110] proposed a deep learning method based on reinforcement learning (RL) [111] to help derive specific DBS patterns, which were able to provide effective DBS controllers and energy efficiency. This RL-based method was evaluated on a brain-on-a-chip field-programmable gate array (FPGA) [112] platform to conduct the basal ganglia model (BGM) [113].

In general, the amount of data obtained from a single brain-on-a-chip is limited. However, the fact that the manufacturing processes of a brain-on-a-chip and a brain organoid-on-a-chip can be labor-intensive and time-consuming [114], makes it difficult to introduce high-throughput analysis or deep learning in some scenarios.

### 4.6. Kidney-on-a-Chip

The kidney is an important excretory organ responsible for maintaining osmotic pressure and the internal environment. Kongadzem et al. [115] reported that the kidney-on-a-chip can be used to overcome the shortcomings of traditional animal models and perform the following operations: first, improving the dosages of drugs in kidney diseases. Second, using the kidney-on-a-chip can help understand the increase in blood urea and other nitrogenous waste. In addition, the kidney-on-a-chip can help in drug testing and development for kidney diseases so as to more effectively identify the drug efficacy, drug-induced nephrotoxicity, and interactions.

**Figure 12:**
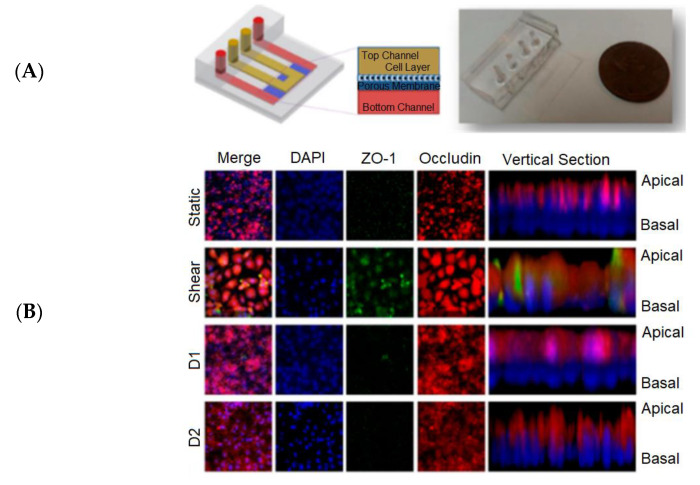
The kidney-on-a-chip was developed for monitoring nephrotoxicity [116]. (**A**) Schematic and actual image of the kidney-on-a-chip. (**B**) Biomarker expressions by the cells in the kidney-on-a-chip in different groups. Reproduced with permission from MDPI Copyright (2023).

Kim et al. [116] reported a pharmacokinetic profile that could reduce the nephrotoxicity of gentamicin in a perfused kidney-on-a-chip platform (Figure 12A), which provided the structure of a kidney-on-a-chip and junctional protein expression of each group. In Figure 12B, the static and shear groups were measured before exposure to gentamicin, and D1 and D2 groups were measured 24 h after exposure to gentamicin. Compared with the Transwell cultures, the polarization of all groups was improved.

Since the activities and mechanics of a kidney can be stimulated by the kidney-on-a-chip, it is expected that the developed chip can function as a normal kidney component for conducting effective drug testing [115]. This will generate a large amount of data because it is necessary to determine the parameter values required for drug efficacy from the cell measurements in the kidney-on-a-chip. Deep learning can analyze these parameters in order to classify or predict the cell response to drugs in the chip and then determine the drug efficacy.

Nowadays, drug-induced kidney injury (DIKI) is one of the leading causes of failure of drug development programs in the clinic. Early prediction of the renal toxicity potential of drugs is crucial to the success of drug candidates in the clinic. The development of kidney-on-a-chip technology is crucial to improve the early prediction of DIKI [73]. Kulkarni et al. [117] reported that newer in silico and computational techniques, such as physiologically based pharmacokinetic modeling and machine learning, have demonstrated potential in assisting the prediction of DIKI. Several machine learning models, such as random forest, support-vector machine, j-nearest neighbor, naïve Bayes, extreme gradient boost, regression tree, and others, have been studied for the prediction of kidney injury [70,71,72]. Machine learning may improve the DIKI predictive ability of the biomarker by automatically identifying non-linear decision boundaries and classifying compounds as toxic or nontoxic with greater accuracy [72]. Potentially, the kidney-on-a-chip can simulate certain functions of a kidney, and deep learning is more suitable for tackling massive data than machine learning. Therefore, the progress in kidney-on-a-chip platforms, in combination with the ability of deep learning, can be a new alternative for resolving DIKI in the future.

### 4.7. Skin-on-a-Chip

**Figure 13:**
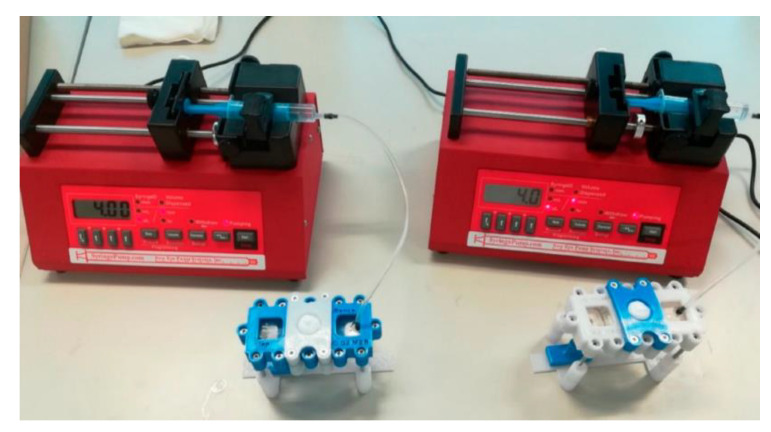
The experimental setup consists of two simultaneous skins-on-a-chip [119]. This setup contains a flow-through dynamic microfluidic device and a programmable syringe pump. The experimental samples can be collected below the diffusion system in the collection bench. Reproduced with permission from MDPI Copyright (2023).

When the skin contacts the external environment, ultraviolet rays, pollutants, and microorganisms in the environment can cause skin diseases [118]. In recent years, drug delivery through the skin has also become a research hotspot, including the screening of drugs in vitro by using the skin-on-a-chip. This miniaturized chip based on microfluidics is a platform to mimic the skin and its equivalents in a simple manner. Figure 13 depicts a solution for designing the skin-on-a-chip for testing drug penetration through the skin [119].

Sutterby et al. [74] reported that the skin-on-a-chip circumvented the drawbacks of traditional cell models by imparting control in the microenvironment and inducing related mechanical information. The skin-on-a-chip assesses the metabolic parameters (O_2_, pH, and glucose and lactate) via embedded microsensors so as to assist in the rigorous evaluation of cell health and streamline the drug testing process. This process has the potential to be intelligentized since the various metabolic parameters can provide multi-source labeled datasets for training a deep network. A possible solution for this is to learn a mapping between these metabolic parameters and their labels through deep learning so as to classify the cells as healthy or unhealthy. In this way, deep learning can further improve the prediction accuracy of drug absorption rate through the skin.

## 5. Discussion

Recently, researchers in different fields have started trying to solve problems in their respective fields with deep learning. Some reports show that the integration of OOCs and deep learning has broad prospects, which can further extend to developing patients-on-a-chip for precision medicine [120]. Meanwhile, there are also various challenges in the future applications of deep learning [121].

### 5.1. Upcoming Technical Challenges

Data with automatic annotation. The development of automatic data annotation algorithms and tools can automatically label a large number of unlabeled data, reduce the tremendous cost of manual annotation, and enhance the efficiency of annotation and development [122]. The automatic data annotation algorithms and tools can effectively expand training and validation datasets so as to improve the prediction accuracy of the neural networks, which are trained for classifying single-cell trajectories, tracking, and motion analyses of cell clusters and particles in time-lapse microscopy images.

Automated network design. As an important branch of AutoML [123], neural architecture searching (NAS) [124] has attracted more and more attention. In deep learning-based tasks of classification, detection, segmentation, and tracking, the structure of the neural network has a decisive impact on the performance of the overall algorithm. The traditional structure designs of neural networks require expert knowledge and trial-and-error costs. Therefore, it is extremely difficult to manually design network structures. The NAS tries to automatically design a network structure with good performance and fast computing speed and frees people from complex network tuning. The ideal NAS technology only requires a user-defined dataset, and the entire system can try various network structures and network connections. Through training, optimizing, and modifying these neural networks, the system gradually outputs a desired network model. The NAS methods replace the conventional time-consuming process by avoiding “manual design-try-modify-try”. There are two main challenges during network design: intractable search space and non-transferable optimality. Different from the hyperparameter optimization (HO) [125] for network training, the NAS is adopted to optimize the parameters that define the network structure.

Multi-variate time-series. The analysis of short-term cardiovascular time series can help to achieve the early detection of cardiovascular diseases. Integrated AI systems can help expedite time-series analysis and improve the accuracy of time-series prediction. The key models for time-series data in computer science (such as NLP) are sequence-to-sequence (seq2seq) models [126], attention models [127], transformer models [128], and graph neural networks (GNN) [129]. These technologies can help explore the relationship network and correlation weights between different data points to increase the accuracy of prediction and analysis. The seq2seq-based time-series anomaly detection methods can detect abnormal fragments in cardiovascular time series. Attention models generally are utilized in neural network models for sequence prediction, which makes the model pay more attention to the relevant parts of historical variables and current input variables. TPA-LSTM [130] is one of the multi-variate time series forecasting approaches, and it modifies the conventional attention mechanism by paying more attention to the selected important, relevant variables rather than all relevant variables. Conventional multi-variate time-series anomaly detection has the following challenges, such as a large amount of data and the requirement of real-time ability. The transformer is a seq2seq model using the self-attention mechanism, and its advantage is the ability of parallel computing. Based on this advantage, the transformer can conduct quick anomaly detection in a large amount of multi-variate time series over a wide time span. Moreover, the multi-variate time series requires additional technologies to handle the issue of high dimensions, especially to capture the potential relationships between dimensions. The introduction of GNN is a way to model spatial dependencies or the relationship between dimensions. The survey [131] demonstrates that the combination of GNN and attention model/transformer can significantly improve the performance of multi-variate time-series prediction. Therefore, using the transformer and GNN to model multi-variate time-series data is worth further studying. In addition, multimodal input data [132,133] (e.g., statistical data of cardiovascular time series, text data of subjective physician’s experience, and image of electrocardiogram) can further perfect the performance of a multi-variate time-series analysis system.

### 5.2. Promising Applications

**Figure 14:**
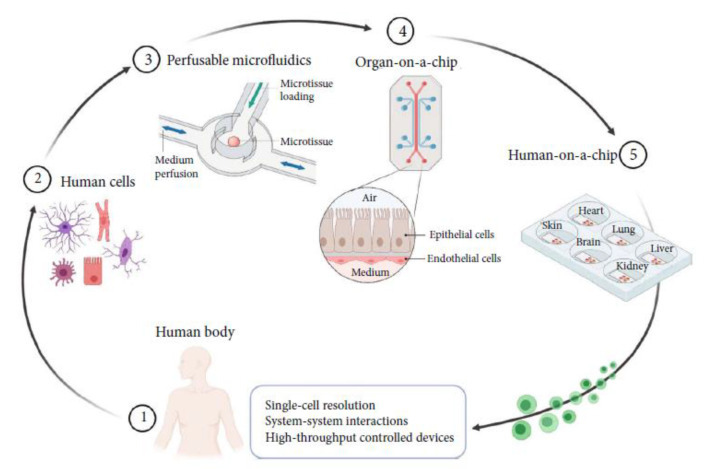
Extracted cells (2) from a human body (1) are placed in perfusable microfluidics (3) to construct OoCs (4). Multiple OoCs are combined in a human-on-a-chip (5) [2]. Reproduced with permission from American Association for the Advancement of Science Copyright (2023).

Human-on-a-chip. As shown in Figure 14, a human-on-a-chip consists of multiple OoCs with different organ representations [2]. Future works can possibly focus on analyzing multi-scale data of each OoC (e.g., the growth, differentiation, or metabolism of cells) and their interactions by using deep learning methodologies so as to integrate OoCs as fully controllable microfluidic platforms and achieve high-throughput assays at single-cell resolution.

Rare disease-on-a-chip. Although OoCs have achieved significant progress in in vitro disease models, drug development for rare diseases is greatly hindered due to a lack of appropriate preclinical models for clinical trials [134,135]. Building rare diseases-on-a-chip can generate important real-time datasets, which is hardly observable in clinical or in vivo samples [136]. Such datasets can be utilized to train a deep learning model for analyzing the changes of such rare diseases at the molecular level and further study the mechanisms of disease occurrence, along with improved capacities in drug discovery by conducting larger-scale clinical trials on OoCs not possible with small pools of patients.

## Figures and Tables

**Table 1:** Summary of different applications of deep learning used for OoCs.

Network	Platform	Function	Refs
CNN	OoC	Improve the spatial resolution of TLM videos for observing cell dynamics and interactions.	[41]
GAN	OoC	Providing high-throughput videos with more cell content for accurately reconstructing cell-interaction dynamics.	[43]
CNN	OoC	Segment nerve cell images into axons, myelins, and background.	[44]
AlexNET	OoC	Classify the treated cancer cells and untreated cancer cells according to their trajectories.	[54]
NN	Lung-on-a-chip	Predict the toxicity for drug discovery via image analysis.	[67]
GAN, CNN	Gut-on-a-chip	Enhance the resolution of confocal fluorescence photographs and conduct a better analysis of protein expression.	[68]
CNN, RNN	Brain-on-a-chip, Brain organoid-on-a-chip	Read the data for analysis in both HCS and HTS via deep learning rather than in a labor-intensive manner.	[69]
CNN	Kidney-on-a-chip	Improve early prediction of DIKI.	[70,71,72,73]
CNN	Skin-on-a-chip	Classify the skin cells as healthy or unhealthy based on metabolic parameters acquired from sensors.	[74]

## Data Availability

Not applicable.
